# Aroylketene dithioacetal chemistry: facile synthesis of 4-aroyl-3-methylsulfanyl-2-tosylpyrroles from aroylketene dithioacetals and TosMIC

**DOI:** 10.1186/1860-5397-3-31

**Published:** 2007-09-28

**Authors:** H Surya Prakash Rao, S Sivakumar

**Affiliations:** 1 Department of Chemistry, Pondicherry University, Puducherry, 605 014, India

## Abstract

The cycloaddition of the von Leusen's reagent (*p*-tolylsulfonyl)methyl isocyanide (TosMIC) to α-aroylketene dithioacetals (AKDTAs) in the presence of sodium hydride in THF at rt resulted in a facile synthesis of the 4-aroyl-3-methylsulfanyl-2-tosylpyrroles **3** in good yield along with a minor amount of 4-[(4-methylphenyl)sulfonyl]-1-[(methylsulfanyl)methyl]-1*H*-imidazole **4**. This study lead to the synthesis of 2,3,4-trisubstiuted pyrroles having 1-naphthoyl/2-naphthoyl/ferrocenoyl/pyrenoyl scaffolds on C-4 of the pyrrole ring. In this transformation, the AKDTAs behave as a 3-(methylsulfanyl)-1-aryl-2-propyn-1-one (ArCOC≡CSMe) equivalent. Competition experiments clearly showed that the cycloaddition of TosMIC to "push-pull" alkenes like AKDTAs is slower compared to general α,β-unsaturated carbonyl compounds.

## Background

The α-aroylketene dithioacetals (AKDTAs) **1** are useful three-carbon synthones extensively employed for the synthesis of a wide variety of heterocyclic compounds and also in several aromatic ring annulation reactions. [1–2] The AKDTAs are α,β-unsaturated carbonyl compounds with two electron-donating alkylsulafanyl groups on one end and an electron-withdrawing aroyl group at the other end of the double bond, i.e., they are "push-pull" alkenes. Depending on the nucleophile and the reaction conditions either 1,2- or 1,4-necleophilc additions on **1** are possible. [3] Since alkylsulfanyl groups are good leaving groups, subsequent to the attack of a nucleophile, one of the alkylsulfanyl groups of the intermediate leave to regenerate the conjugated system. Being polarized alkenes the AKDTAs also react with bi-functional molecules having nucleophilic and electrophilic centers to furnish cyclic compounds. We have recently synthesized a combinatorial library of 3-aroylcoumarins by the reaction of 2-hydroxyarylaldehydes and the AKDTAs in presence of a catalytic amount of piperidine in THF. [4] Notwithstanding the popularity of AKDTAs in the synthesis of heterocyclic systems, examples of their participation in the cycloaddition reactions are rare. In continuation of our interest on ketene acetals [5–7] we considered that 1,3-dipolar cycloaddition of the anion generated from the von Leusen's reagent (*p*-tolylsulfonyl)methyl isocyanide (TosMIC) **2** to AKDTAs **1** could lead to 2,3,4-trisubstituted pyrroles having an aroyl moiety located on C-4. Von Leusen's and others have amply demonstrated that the cycloaddition of TosMIC to the alkenes having strong electron-withdrawing groups (Michael acceptors) followed by loss of TosOH provide 3,4-disubstituted pyrroles in excellent yield. [8–10] Similarly, the reaction of TosMIC with activated alkynes afford trisubstituted pyrroles. However, the scope of pyrrole synthesis via cycloaddition of TosMIC to the "push-pull" alkenes is not well explored. We reasoned that, in the event of cycloaddition taking place between TosMIC and AKDTAs, the intermediate from the reaction would have three good leaving groups, namely, two methylsulfanyl and a tosyl. The release of one of the methylsulfanyl group could afford trisubstituted pyrroles having C-4 aroyl group. In such an outcome, the AKDTA substrates behave as a 3-(methylsulfanyl)-1-aryl-2-propyn-1-one (ArCOC≡CSMe) equivalent. We report herein realization of the idea for the synthesis of trisubstituted pyrroles **3** from AKDTAs **1** and TosMIC **2**. The pyrrole is one of the fundamental heterocycles. Its structure is widely found in many biological molecules. Naturally, there is a continuous interest to develop new synthesis of pyrroles. [11–13] Moreover, the target molecules of the present study, namely, 2,3,4-trisubstiuted pyrroles incorporate cannabinoid 3-aroylpyrrole structural motif [14] and that of some photo activators. [15] A recent report from Ila and co-workers on the use of some isocyanides for the syntheses of trisubstituted pyrroles prompts us to disclose our efforts in this area. [16]

## Results and Discussion

**Scheme 1:**
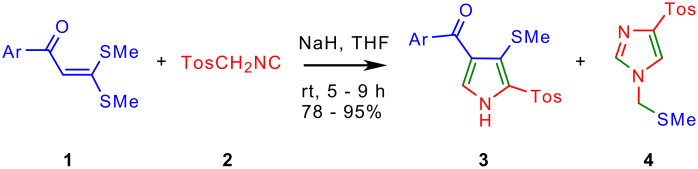
Cycloaddition of TosMIC to AKDTAs provide 2,3,4-trisubstituted pyrroles **3** and imidazole **4**.

The cycloaddition of TosMIC **2** to 3,3-di(methylsulfanyl)-1-phenyl-2-propen-1-one **1a** was taken as a test case to evaluate the cycloaddition and to arrive at the optimal reaction conditions. The cycloaddition of **2** to **1a** took place smoothly in the presence of NaH in THF at rt to furnish [5-[(4-methylphenyl)sulfonyl]-4-(methylsulfanyl)-1*H*-3-pyrrolyl](phenyl)methanone **3a** in 92% yield (Scheme 1 and Table 1). The cycloaddition did not take place when piperidine in THF or K_2_CO_3_ in acetone reflux was employed. However, it is to be noted that when Ila and coworkers employed NaH/THF for the cycloaddition of ethyl isocyanoacetate to AKDTA **1a** only 20% yield of the desired cycloadduct was obtained. [16] Near quantitative yield (92%) in our reaction indicates higher reactivity of NaH/THF with TosMIC. Two singlets at δ 2.23 and δ 2.42 ppm in the ^1^H NMR spectrum of **3a** assignable for methyls of SMe and tosyl groups respectively served as diagnostic signals. As anticipated the ^13^C NMR spectrum of **3a** displayed fifteen signals, with a diagnostic signal at δ 127.9 ppm assignable to pyrrole C-5.

**Table 1:** Synthesis of pyrroles 3 from AKDTAs **1** and TosMIC **3**

Entry	Ar	AKDTA **1**	Pyrrole **3**	Time (h)	Yield (%) of **3**^a^	Yield (%) of **4**^b^

1	C_6_H_5_	**1a**	**3a**	8	92	3
2	4-CH_3_C_6_H_4_	**1b**	**3b**	8	90	4
3	4-ClC_6_H_4_	**1c**	**3c**	6	87	5
4	4-C_6_H_5_C_6_H_4_	**1d**	**3d**	5	93	3
5	1-C_10_H_7_	**1e**	**3e**	5	87	4
6	2-C_10_H_7_	**1f**	**3f**	6	85	5
7	C_5_H_5_FeC_5_H_4_	**1g**	**3g**	9	78	12

^a^ yield is based on the AKDTAs used. ^b^ yield is based on TosMIC used.

**Scheme 2:**
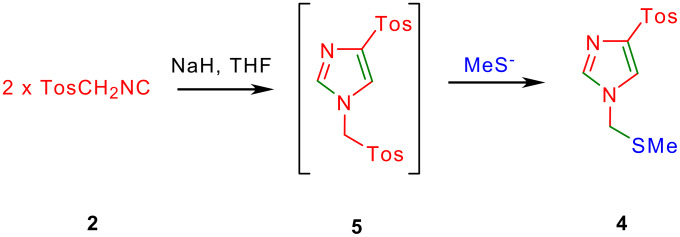
Base induced dimerization of TosMIC to furnish imidazole **4**.

Careful examination of the reaction mixture from the reaction of TosMIC with **1** revealed the presence of a minor product which was characterized as 4-[(4-methylphenyl)sulfonyl]-1-[(methylsulfanyl)methyl]-1*H*-imidazole **4** on the basis of its spectral and analytical data (Scheme 1 and Table 1). Previously, van Leusen's studied the base induced decomposition of TosMIC in K_2_CO_3_ in MeOH-DME or NaH in DME and found that one of the decomposition product was 4-[(4-methylphenyl)sulfonyl]-1-[(4-methylphenyl)sulfonyl]methyl-1*H*-imidazole **5** (Scheme 2). [17–18] The imidazole **4** isolated in the present study could arise by substitution of tosyl group in **5** with methylsulfanyl group by in situ nucleophilic attack of methylsulfanyl anion. Yield of the side-product **4** increased at the cost of the desired pyrroles **3** when the reaction was conducted in THF reflux.

**Figure 1:**
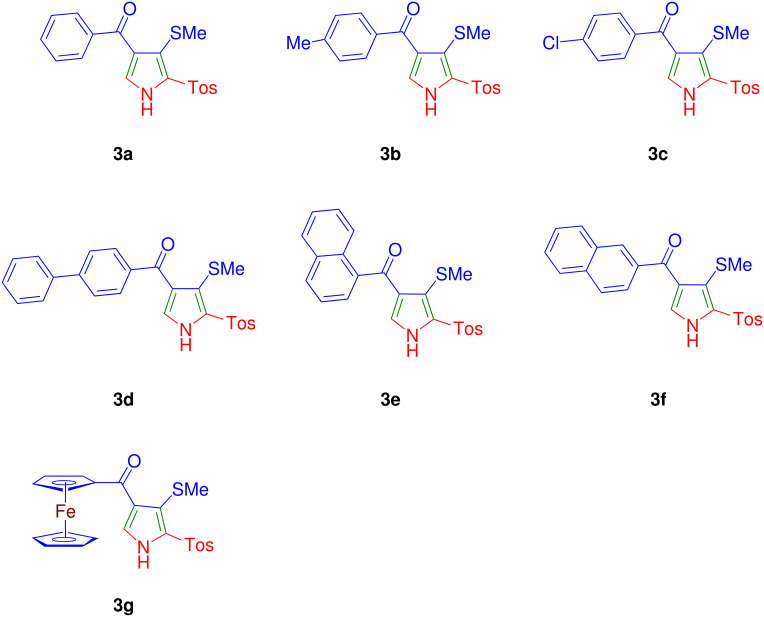
Structure of 2,3,4-trisubstituted pyrroles **3a-g** synthesized in this study.

The cycloaddition of TosMIC **2** to a variety of AKDTAs **1b**-**g** furnished good yield of new pyrroles **3b**-**g** in a facile manner, which demonstrated generality of the transformation (Scheme 1, Table 1 & Figure 1). The analytical and spectral data of **3b**-**g** compared well with the parent pyrrole **3a**. In each case, desired pyrroles **3b**-**g** were separated from minor amount of the imidazole **4** contaminant by column chromatography. The structure of **3b** was confirmed on the basis of the single crystal X-ray structure analysis (deposited with Cambridge Crystallographic Data Center (CCDC); deposition No. 628766). The structure of AKDTAs **1b**-**g** appears to have no major influence on the observed yield of the pyrroles **3**. The AKDTAs having electron donating (4-Me, **1b** or ferrocene **1g**), or electron-withdrawing groups (4-Cl, **1c** or 4-Ph, **1d**) furnished corresponding pyrroles in good and comparable yield. The 2,3,4-trisubstituted pyrroles having 4-phenylbenzoyl (**3d**), 1-naphthoyl (**3e**), 2-naphthoyl (**3f**) and ferrocenoyl (**3g**) groups are to be noted for the variety of pyrrole derivatives prepared (Figure 1). It is interesting to note that [5-[(4-methylphenyl)sulfonyl]-4-(methylsulfanyl)-1*H*-3-pyrrolyl](1-naphthyl)methanone **3e** has the structural motif of some of the cannabinoids. [14]

**Scheme 3:**
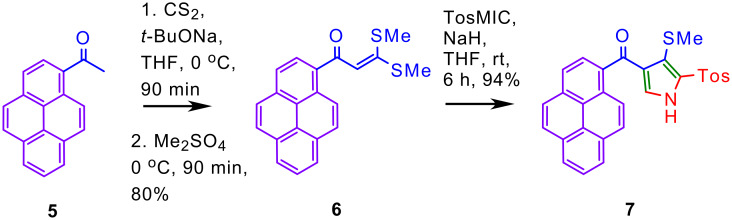
Synthesis of AKDTA **6** and it's further transformation to pyrrole **7**.

In continuation, and by following the method presently developed, the trisubstituted pyrrole **7** having pyrenoyl substituent was synthesized from AKDTA **6** (Scheme 3). Initially, 1-acetylpyrene **5** was transformed to hitherto unknown pyrene based AKDTA **6** by reaction with carbon disulfide and sodium *tert*-butoxide followed by alkylation with dimethyl sulfate. The reaction of TosMIC **2** with AKDTA **6** provided the trisubstituted pyrrole **7** in excellent yield. As anticipated, the ^1^H NMR spectrum of pyrenoylpyrrole **7** exhibited a doublet for C2'-H of pyrene unit as a doublet at δ 8.37 ppm and a singlet at δ 7.06 ppm for C5-H of the pyrrole unit.

**Scheme 4:**
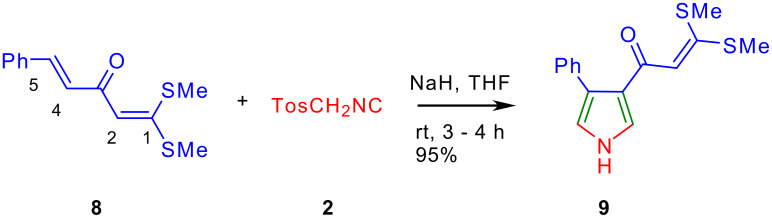
Regio-selectivity in the reaction of TosMIC with **8**.

**Scheme 5:**
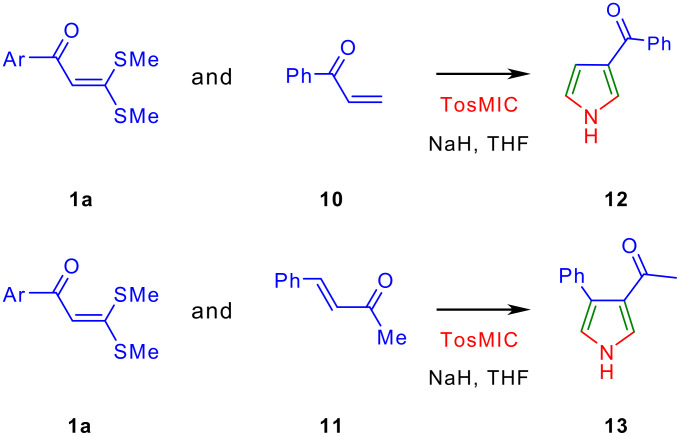
Competition experiments involving one mmol each of TosMIC with AKDTA **1a** and phenyl vinyl ketone **10** or with AKDTA **1a** and (*E*)-4-phenyl-3-buten-2-one **11** to provide pyrroles **12** and **13** respectively.

Next we have studied the cycloaddition reaction of TosMIC to (4*E*)-1,1-di(methylsulfanyl)-5-phenyl-1,4-pentadien-3-one **8** [19] a molecule having two double bonds – one between C-1 and C-2 is flanked by the two electron-donating methylsufanyl and the electron-withdrawing carbonyl group and the other one between C-4 and C-5 is flanked by the electron-withdrawing carbonyl and phenyl groups (Scheme 4). The former double bond is a "push-pull" type and is more polarized compared to the latter double bond. This reaction provided 3,3-di(methylsulfanyl)-1-(4-phenyl-1*H*-3-pyrrolyl)-2-propen-1-one **9** exclusively (Scheme 3). Above reaction demonstrated that TosMIC **2** reacts with a double bond flanked by two electron-withdrawing groups in preference over the "push-pull" double bond with electron donating and withdrawing groups. To substantiate the reasoning, competitive cycloaddition experiments between 1 mmol each of AKDTA **1a** and phenyl vinyl ketone **10** or AKDTA **1a** and (*E*)-4-phenyl-3-buten-2-one **11** with 1 mmol of TosMIC **2** were conducted in the presence of NaH in THF and in each case known pyrroles **12** [20] and **13** [21–22] were obtained exclusively (Scheme 5).

## Conclusion

In summary, we reported a facile synthesis of 2,3,4-trisubstituted pyrroles having a variety of aroyl groups in C-4 position from AKDTAs and TosMIC. The competition experiments demonstrated that TosMIC reacts with electron deficient olefins in preference to "push-pull" alkenes. Future works will concentration on desulfuration of the methylsulfanyl group in **3** to generate 3,5-disubstituted pyrroles or oxidation of methylsulfanyl group to methylsulfonyl group followed by nucleophilic displacement to generate pyrroles of structural diversity.

## Experimental

"See the Supporting Information File 1 for the full experimental data"

## Supporting Information

File 1 Supporting information of Experimental procedures and NMR spectra (^1^H and ^13^C) of all the new compounds.
