# Supplementary material for: Aroylketene dithioacetal chemistry: facile synthesis of 4-aroyl-3-methylsulfanyl-2-tosylpyrroles from aroylketene dithioacetals and TosMIC
Source: Beilstein J Org Chem. 2007 Sep 28;3:31. doi: 10.1186/1860-5397-3-31 (PMC2176056; doi:10.1186/1860-5397-3-31)
Supplement: File 1 — Supporting information of Experimental procedures and NMR spectra (1H and 13C) of all the new compounds. [file Beilstein_J_Org_Chem-03-31-s001.doc]

**Aroylketene dithioacetal chemistry: Facile synthesis of 4-aroyl-3-methylsulfanyl-2-tosylpyrroles from aroylketene dithioacetals and tosmic**

H. Surya Prakash Rao^*^ and S. Sivakumar

Department of Chemistry, Pondicherry University, Puducherry – 605 014 INDIA

Fax +91 413 2655987; E.mail: [hspr@yahoo.com](mailto:hspr@yahoo.com)

# SUPPORTING INFORMATION

**Experimental Section**

*General information*: The progression of all the reactions was monitored by TLC using hexanes (60 – 80 ^o^C boiling mixture) / ethyl acetate as eluent. Column chromatography was carried on silica gel (100 – 200 mesh) using increasing percentage of ethyl acetate in hexanes. The ^1^H NMR (300 MHz) or ^13^C NMR (75 MHz) and DEPT spectra were recorded in CDCl_3_ using Brucker 300 MHz or JEOL 60 MHz spectrometers with TMS (0 ppm) as the internal standard. The HRMS (ESI-MS) data were collected by Waters Micromass Q-Tof Micro Quadrapole -Time of Flight Tandem Mass Spectrometer. The IR spectra were recorded as KBr solutions using BOMEM MB 104 FT-IR (ZnSe optics) spectrometer. Melting points were recorded using open-ended capillary tubes and are uncorrected.

**3,3-Di(methylsulfanyl)-1-(1-pyrenyl)-2-propen-1-one 6**: To a stirred suspension of freshly prepared sodium *tert*-butoxide (1.47 g, 15 mol) in dry THF (10 mL) at 0 ºC a solution of 1-acetylpyrene [23] (1 g, 4.0 mmol) and carbon disulfide (1.21 g, 0.96 mL, 8.0 mmol) in dry THF (10 mL) was added through a pressure equalizer funnel and the mixture was vigorously stirred at 0 ºC for 90 min. Appearance of a reddish solid in the reaction medium indicated the formation of disodium 3-oxo-3-(1-pyrenyl)-1-propene-1,1-dithiolate. A solution of dimethyl sulphate (1 g, 0.89 mL, 8 mmol) in dry THF (5 mL) was carefully added to this suspension, drop-wise during 10 min at 0 ^o^C and the reaction mixture was allowed to stir at 0 ^o^C for 90 min. After completion of the reaction (TLC; hexanes: EtOAc = 8:2), the mixture was transferred into a 100 mL beaker containing 50 g of crushed ice and the contents of the beaker were stirred well. A light yellow colored solid formed was filtered and washed with water (20 mL 🞨 3). The crude solid was re-crystallized from EtOH to furnish 1.10 g of 3,3-di(methylsulfanyl)-1-(1-pyrenyl)-2-propen-1-one in 80% yield as light yellow colored crystals; mp 112-115 ºC. IR (KBr) 2945, 1604, 1473, 1375, 1222, 1130, 837, 702 cm^-1^. ^1^H NMR (400 MHz, CDCl_3_): δ 2.44 (s, 3H), 2.57 (s, 3H), 6.70 (s, 1H), 7.9-8.2 (m, 8H), 8.74 (d, *J* = 9.6 Hz 1H). ^13^C NMR (100 MHz, CDCl_3_): δ 15.0 (CH_3_), 17.3 (CH_3_), 114.2 (CH), 124.1 (CH), 124.4 (C), 124.8 (C), 124.9 (CH), 125.5 (CH), 125.6 (CH), 125.7 (CH), 126.2 (CH), 127.1 (CH), 128.5 (CH), 128.7 (CH), 128.8 (C), 130.7 (C), 131.1 (C), 132.7 (C), 135.7 (C), 165.9 (C), 189.6 (C=O). HRMS (ESI^+^) calcd for C_21_H_16_OS_2_ (MH^+^) 349.0643; found 349.0623.

**Synthesis of 2,3,4-trisubstituted pyrroles 3a-g and 7; General procedure**

To a stirred and cleaned suspension of NaH (50% suspension in oil; 48 mg, 2.0 mmol) in dry THF (5 mL) at 0 °C was added a mixture of TosMIC **2** (235 mg, 1.2 mmol) and α-aroylketene dithioacetal **1a-g** or **6**(1.0 mmol) in dry THF (10 mL) during 30 min. The reaction mixture was then allowed to stir at rt for 5 - 9 h. After completion of the reaction (TLC), the mixture was carefully acidified with acetic acid up to pH 6.5 followed by dilution with dichloromethane (40 mL). The organic solution was washed with water (3 × 25 mL) and brine (2 × 10 mL) and dried over anhydrous Na_2_SO_4_. Evaporation of the solvent under reduced pressure resulted in the trisubstituted pyrroles **3a-g** or **7** along with minor amounts of the imidazole **4**. The crude product was subjected to column chromatography on SiO_2_ using increasing amounts of ethyl acetate in hexanes (60 - 80 ºC boiling mixture) as eluent. Evaporation of the pooled fractions having the required trisubstituted pyrroles **3a-g** or imidazole **4** resulted in crystalline products. Re-crystallization using EtOH furnished the analytically pure samples.

**[5-[(4-Methylphenyl)sulfonyl]-4-(methylsulfanyl)-1*H*-3- pyrrolyl](phenyl)**

**methanone 3a**: Yield: 92%; mp 114-116 ºC (DCM / hexanes). IR (KBr): 3284, 1649, 1594, 1515, 1436, 1353, 1329, 1218, 1147, 1072, 961, 729, 694, 655 cm^-1^. ^1^H NMR (300 MHz, CDCl_3_): δ 2.23 (s, 3H), 2.42 (s, 3H), 7.2–7.3 (m, 3H), 7.43 (t, *J* = 7.8 Hz, 2H), 7.54 (t, *J* = 7.8 Hz, 1H), 7.75 (d, *J* = 6.9 Hz, 2H), 7.98 (d, *J* = 9.8 Hz, 2H), 10.46 (br s, 1H). ^13^C NMR (75 MHz, CDCl_3_): δ 19.85 (CH_3_), 21.65 (CH_3_), 122.04 (C), 127.94 (CH × 2), 128.24 (CH), 128.32 (CH × 2), 129.31 (CH × 2), 129.67 (CH × 2), 132.34 (C), 132.40 (CH), 137.68 (C), 138.80 (C), 144.87 (C), 190.06 (C). HRMS (ESI^+^) calcd for C_19_H_17_NO_3_S_2_ (MH^+^) 372.0729; found 372.0703.

**4-[(4-Methylphenyl)sulfonyl]-1-[(methylsulfanyl)methyl]-1*H*-imidazole (4)**: Yield: 3-12%; mp 150 ºC (DCM / hexanes). IR (KBr): 3111, 1515, 1420, 1303, 1151, 1119, 1041, 810, 704, 596 cm^-1^. ^1^H NMR (400 MHz, CDCl_3_): δ 2.08 (s, 3H), 2.41 (s, 3H), 4.97 (s, 2H), 7.32 (d, *J* = 6.7 Hz, 2H), 7.79 (s, 1H), 7.94 (d, *J* = 6.7 Hz, 2H). ^13^C NMR (100 MHz, CDCl_3_): δ 4.67 (CH_3_), 21.60 (CH_3_), 50.97 (CH_3_), 115.20 (CH_3_), 122.98 (C), 128.04 (CH), 129.81 (CH), 137.48 (C), 138.74 (CH), 142.38 (CH), 144.42 (C). HRMS (ESI^+^) calcd for C_12_H_14_N_2_O_2_S_2_ (MH^+^) 283.0579; found 283.2315.

**(4-Methylphenyl)[5-[(4-methylphenyl)sulfonyl]-4-(methylsulfanyl)-1*H*-3-pyrrolyl]methanone (3b)**: Yield: 90%; mp 152 ºC (DCM / hexanes). IR (KBr): 3259, 1644, 1603, 1515, 1429, 1349, 1327, 1220, 1144, 1050, 986, 886 cm^-1^. ^1^H NMR (300 MHz, CDCl_3_): δ 2.22 (s, 3H), 2.41 (s, 3H), 2.42 (s, 3H), 7.23 (d, *J* = 8.1 Hz, 2H), 7.29 (d, *J* = 3.6 Hz, 1H), 7.32 (d, *J* = 3.1 Hz, 2H), 7.68 (d, *J* = 8.1 Hz, 2H), 7.98 (d, *J* = 8.1Hz, 2H), 10.33 (br s, 1H). ^13^C NMR (75 MHz, CDCl_3_): δ 19.97 (CH_3_), 21.58 (CH_3_), 21.63 (CH_3_), 121.86 (C), 127.65 (CH), 127.91 (C), 127.96 (CH × 2), 129.03 (CH × 2), 129.59 (CH × 2), 129.67 (CH × 2), 132.21 (C), 136.07 (C), 137.75 (C), 143.31 (C), 144.84 (C), 189.93 (C). HRMS (ESI^+^) calcd for C_20_H_19_NO_3_S_2_ (MH^+^) 386.5019; found 386.0859.

**(4-Chlorophenyl)[5-[(4-methylphenyl)sulfonyl]-4-(methylsulfanyl)-1*H*-3-pyrrolyl]methanone 3c**: Yield: 87%; mp 152 ºC (DCM / hexanes). IR (KBr): 3111, 1627, 1591, 1519, 1353, 1330, 1219, 1147, 1085, 863, 847 cm^-1^. ^1^H NMR (400 MHz, CDCl_3_): δ 2.20 (s, 3H), 2.43 (s, 3H), 7.26 (s, 1H), 7.33 (d, *J* = 7.8Hz, 2H), 7.41 (d, *J* = 8.3 Hz, 1H), 7.71 (d, *J* = 8.5 Hz, 2H), 7.98 (d, *J* = 8.1 Hz, 2H), 10.23 (br s, 1H) – ^13^C NMR (100 MHz, CDCl_3_): δ 19.85 (CH_3_), 21.65 (CH_3_), 122.04 (C), 127.94 (CH × 2), 128.24 (CH), 128.32 (CH × 2), 129.31 (CH × 2), 129.67 (CH × 2), 132.34 (C), 132.40 (CH), 137.68 (C), 138.80 (C), 144.87 (C), 198.37 (C). HRMS (ESI^+^) calcd for C_19_H_16_NO_3_S_2_Cl (MH^+^) 406.0339; found 406.0313.

**(4-Biphenyl)[5-[(4-methylphenyl)sulfonyl]-4-(methylsulfanyl)-1*H*-3-pyrrolyl]methanone 3d**: Yield: 93%; mp 152 ºC (DCM / hexanes). IR (KBr): 3328, 3303, 1681, 1605, 1508, 1422, 1297, 1142, 749, 695, 600, 503 cm^-1^. ^1^H NMR (300 MHz, CDCl_3_): δ 2.24 (s, 3H), 2.42 (s, 3H), 7.32 (d, *J* = 8.1 Hz, 2H), 7.36 (s, 1H), 7.37 - 7.67 (m, 7H), 7.85 (d, *J* = 8.2 Hz, 2H), 7.80 (d, *J* = 8.2 Hz, 2H), 10.40 (br s, 1H). ^13^C NMR (75 MHz, CDCl_3_): δ 20.40 (CH_3_), 22.11 (CH_3_), 122.41 (C), 127.46 (CH × 2), 127.67 (CH × 2), 128.18 (CH), 128.37 (CH × 2), 128.43 (CH), 129.38 (CH × 2), 130.15 (CH × 2), 130.47 (C), 132.47 (C), 137.84 (C), 138.12 (C), 140.26 (C), 145.36 (C), 145.68 (C), 190.10 (C). HRMS (ESI^+^) calcd for C_25_H_21_NO_3_S_2_ (MH^+^) 448.1039; found 448.1048.

**[5-[(4-Methylphenyl)sulfonyl]-4-(methylsulfanyl)-1*H*-3-pyrrolyl](1-naphthyl)methanone 3e**: Yield: 87%; mp 120-122 ºC (DCM / hexanes). IR (KBr): 3272, 1641, 1590, 1466, 1429, 1348, 1267, 1148, 1084, 780, 680 cm^-1^. ^1^H NMR (300 MHz, CDCl_3_): δ 2.25 (s, 3H), 2.40 (s, 3H), 7.05 (d, *J* = 8.6 Hz, 1H), 7.28 (d, *J* = 8.1Hz, 2H), 7.3-7.6 (m, 4H), 7.8-7.95 (m, 2H), 7.95 (d, *J* = 8.1 Hz, 2H), 8.10 (br d, *J* = 7.2 Hz, 1H), 10.55 (br s, 1H). ^13^C NMR (75 MHz, CDCl_3_): δ 19.41 (CH_3_), 21.60 (CH_3_), 121.96 (C) 124.24 (CH), 125.36 (CH), 126.43 (CH × 2), 127.20 (CH × 2), 127.93 (CH × 2), 128.26 (CH), 129.62 (CH × 2), 130.13 (CH), 130.46 (C), 131.15 (CH), 130.46 (C), 131.15 (CH), 132.58 (C), 133.11 (C), 133.62 (C), 137.70 (C), 141.81 (C), 191.44 (C). HRMS (ESI^+^) calcd for C_23_H_19_NO_3_S_2_ (MH^+^) 422.0884; found 422.0927.

**[5-[(4-Methylphenyl)sulfonyl]-4-(methylsulfanyl)-1*H*-3-pyrrolyl](2-naphthyl)methanone 3f**: Yield: 83%; mp 180 - 182 ºC (DCM / hexanes). IR (KBr): 3284, 1653, 1514, 1308, 1210, 1137, 824, 730, 605 cm^-1^. ^1^H NMR (400 MHz, CDCl_3_): δ 2.23 (s, 3H), 2.41 (s, 3H), 7.30 (d, *J* = 8.4 Hz, 2H), 7.36 (s, 1H), 7.52 (t, *J* = 7.8Hz, 1H), 7.78 (t, *J* = 7.8 Hz, 1H), 7.85-7.90 (m, 4H), 7.99 (d, *J* = 8.0 Hz, 2H), 8.24 (s, 1H), 10.45 (br s, 1H). ^13^C NMR (75 MHz, CDCl_3_): δ 19.96 (CH_3_), 21.61 (CH_3_), 121.98 (C), 125.13 (CH), 126.77 (CH), 127.74 (C), 127.90 (CH), 127.97 (CH × 3), 128.26 (CH), 128.35 (CH), 129.34 (CH), 129.66 (CH), 131.04 (CH × 2), 132.21 (C), 132.40 (C), 135.24 (C), 135.97 (C), 137.70 (C), 144.86 (C), 190.07 (C). HRMS (ESI^+^) calcd for C_23_H_19_NO_3_S_2_ (MH^+^) 422.0884; found 422.0840.

**[5-[(4-Methylphenyl)sulfonyl]-4-(methylsulfanyl)-1*H*-3-pyrrolyl](3-η^5^-ferrocenoyl)methanone 3g**: Yield: 78%; mp 172-174 ºC (DCM / hexanes). IR (KBr): 3365, 1622, 1511, 1378, 1237, 1144, 821, 767, 674 cm^-1^. ^1^H NMR (400 MHz, CDCl_3_): δ = 2.24 (s, 3H), 2.43 (s, 3H), 4.21 (s, 5H), 4.53 (s, 2H), 4.83 (s, 2H), 7.33 (d, *J* = 6.4 Hz, 2H), 7.55 (s, 1H), 8.01 (d, *J* = 7.6 Hz, 2H), 10.11 (br s, 1H). ^13^C NMR (100 MHz, CDCl_3_) δ 20.45 (CH_3_), 21.67 (CH_3_), 70.02 (CH), 70.82 (CH), 72.32 (CH), 79.73 (C), 120.73 (C), 124.58 (CH), 129.67 (C, CH), 132.31 (C), 137.81 (C), 144.81 (C), 192.24 (C). HRMS (ESI^+^) calcd for C_23_H_21_FeNO_3_S_2_ (M + Na^+^) 502.021; found 502.0216.

**[5-[(4-Methylphenyl)sulfonyl]-4-(methylsulfanyl)-1*H*-3-pyrrolyl](1-pyrenyl)**

**methanone 7**: Yield: 94%; mp 142-144 ºC (DCM / hexanes). IR (KBr): 3398, 1654, 1510, 1340, 1145, 906, 850, 665 cm^-1^. ^1^H NMR (400 MHz, CDCl_3_): δ 2.24 (s, 3H), 2.29 (s, 3H), 7.06 (s, 1H), 7.31 (d, *J* = 8.1 Hz, 1H), 7.99 – 8.19 (m, 7H), 8.38 (d, *J* = 9.3 Hz, 1H), 10.08 (s, 1H). ^13^C NMR (100 MHz, CDCl_3_): δ 19.63 (CH_3_), 21.65 (CH_3_), 122.0 (C), 123.7 (CH), 124.0 (CH), 124.5 (C), 124.8 (C), 125.9 (CH), 126.0 (CH), 126.4 (CH), 126.5 (CH), 127.2 (CH), 128.0 (CH), 128.9 (CH), 129.1 (CH), 129.3 (CH), 129.5 (C), 129.7 (CH), 129.9 (C), 130.6 (C), 131.1 (C), 132.7 (C), 133.0 (C), 134.0 (C), 137.7 (C), 144.8 (C), 191.8 (C=O). HRMS (ESI^+^) calcd for C_29_H_21_NO_3_S_2_ (M^+^) 496.0963; found 496.0958.

**3,3-Di(methylsulfanyl)-1-(4-phenyl-1*H*-3-pyrrolyl)-2-propen-1-one 9**: Yield: 95%; mp 148 ºC (DCM/hexanes). IR (KBr): 3324, 3178, 1597, 1488, 1351, 1135, 1094, 1070, 781, 757 cm^-1^. ^1^H NMR (400 MHz, CDCl_3_): δ 1.73 (s, 3H), 2.43 (s, 3H), 6.04 (s, 1H), 6.72 (s, 1H), 7.27 (t, *J* = 6.8 Hz, 1H), 7.3-7.4 (m, 4H), 7.43 (s, 1H), 9.69 (s, 1H). ^13^C NMR (75 MHz, CDCl_3_): δ 14.83 (CH_3_), 15.87 (CH_3_), 114.11 (CH), 118.22 (CH), 124.30 (CH), 124.06 (C), 125.01 (C) 126.51 (CH), 128.09 (CH), 129.57 (CH), 136.30 (C), 161.53 (C), 183.54 (C). HRMS (ESI^+^) calcd for C_15_H_15_NOS_2_ (MH^+^) 290.0674; found 290.2232.


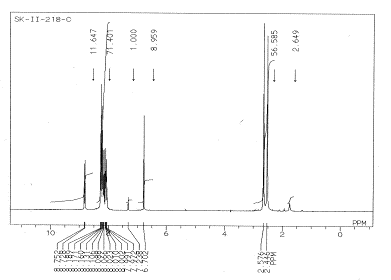


300 MHz (CDCl_3_) ^1^H NMR spectrum of 3,3-di(methylsulfanyl)-1-(1-pyrenyl)-2-propen-1-one **6**


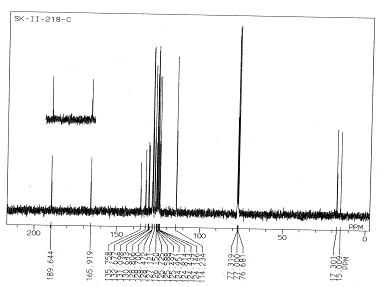


100 MHz (CDCl_3_) ^13^C NMR spectrum of

3,3-di(methylsulfanyl)-1-(1-pyrenyl)-2-propen-1-one **6**


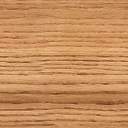


300 MHz (CDCl_3_) ^1^H NMR spectrum of

[5-[(4-methylphenyl)sulfonyl]-4-(methylsulfanyl)-1*H*-3- pyrrolyl](phenyl)methanone **3a**

**]**

75 MHz (CDCl_3_) ^13^C NMR spectrum of

[5-[(4-methylphenyl)sulfonyl]-4-(methylsulfanyl)-1*H*-3- pyrrolyl](phenyl)methanone **3a**

300 MHz (CDCl_3_) ^1^H NMR spectrum of

4-[(4-methylphenyl)sulfonyl]-1-[(methylsulfanyl)methyl]-1*H*-imidazole **4**

100 MHz (CDCl_3_) ^13^C NMR spectrum of

4-[(4-methylphenyl)sulfonyl]-1-[(methylsulfanyl)methyl]-1*H*-imidazole **4**


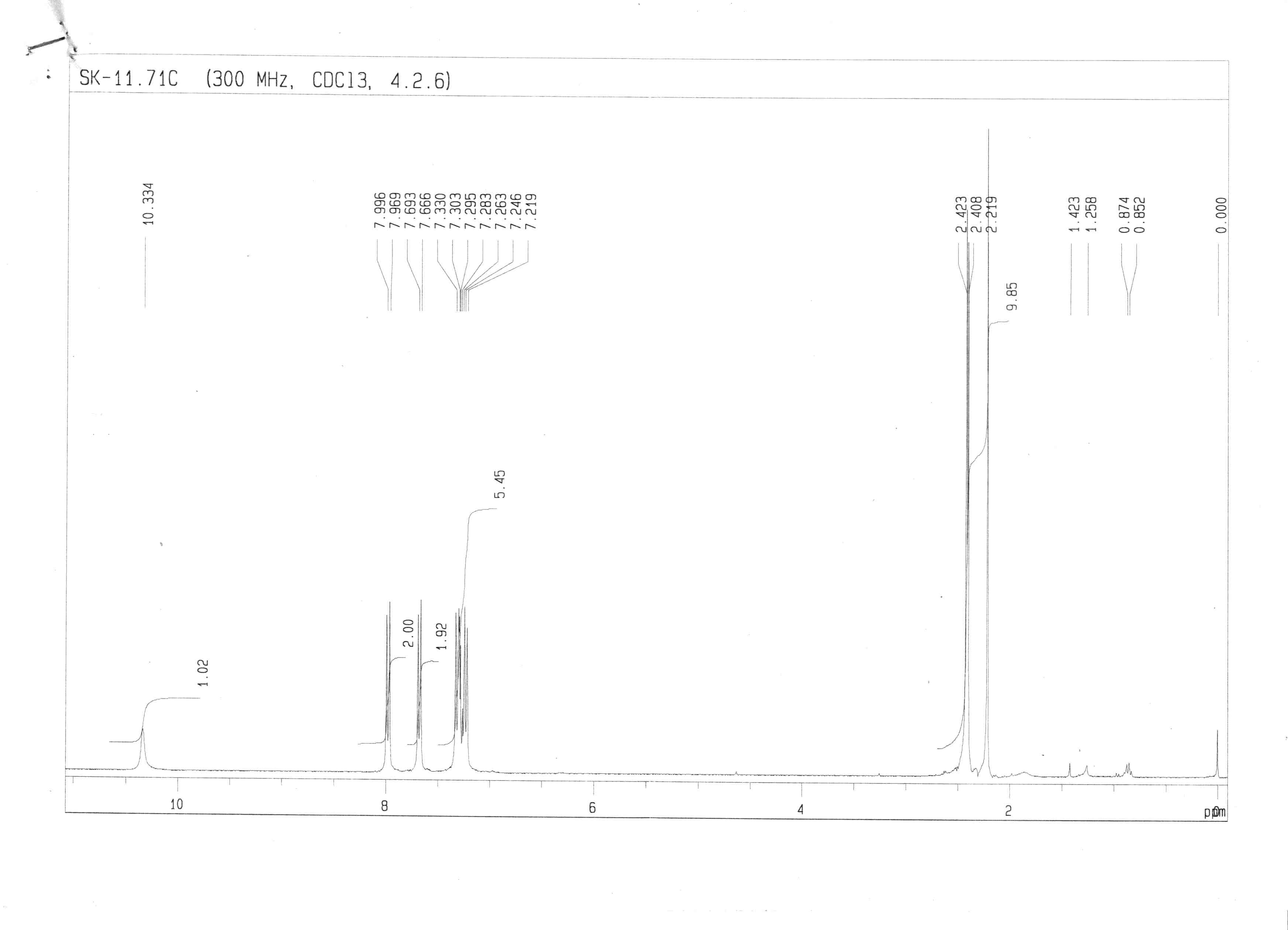


300 MHz (CDCl_3_) ^1^H NMR spectrum of (4-methylphenyl)[5-[(4-methylphenyl)sulfonyl]-4-(methylsulfanyl)-1*H*-3-pyrrolyl]methanone **3b**


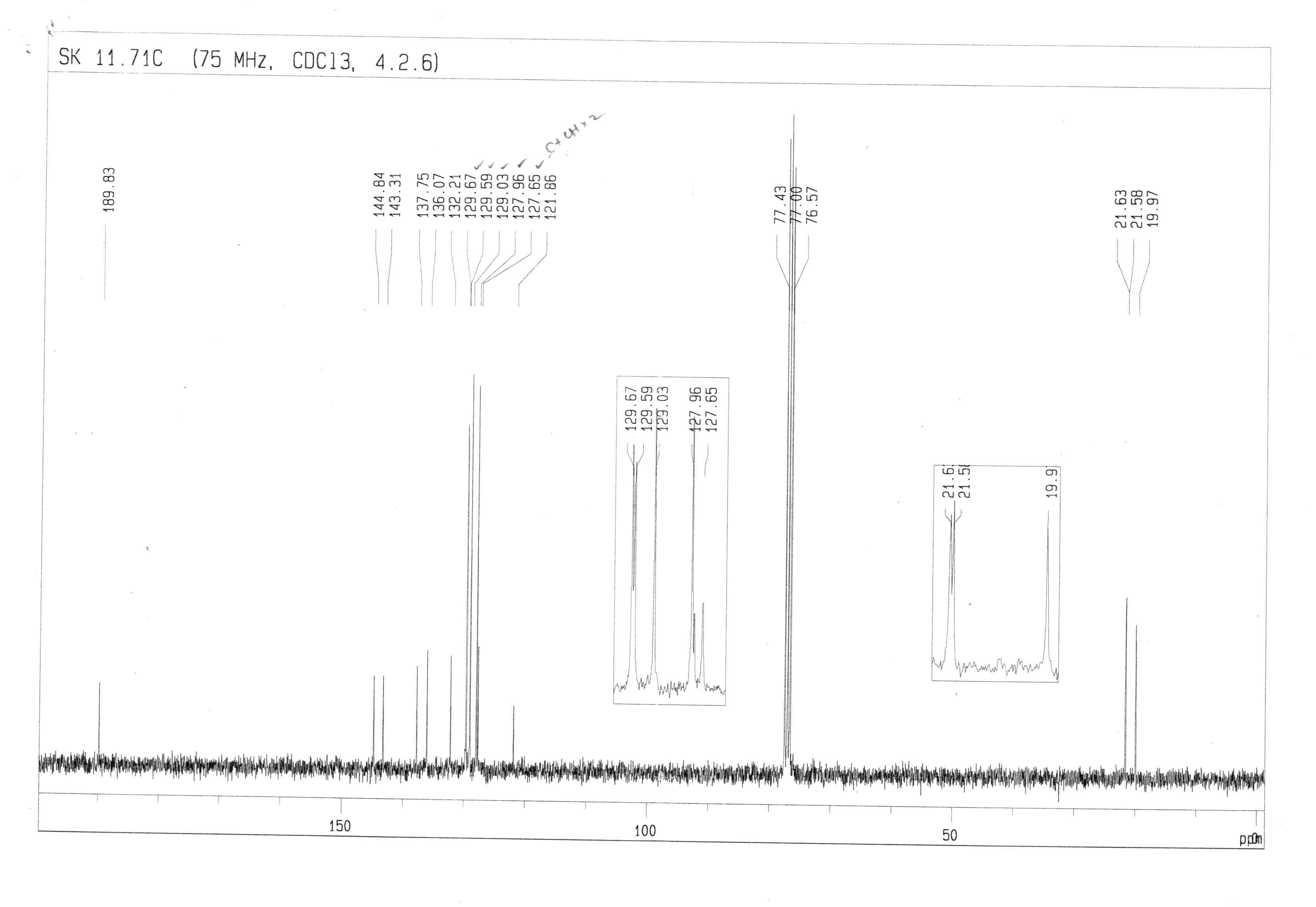


75 MHz (CDCl_3_) ^13^C NMR spectrum of (4-methylphenyl)[5-[(4-methylphenyl)sulfonyl]-4-(methylsulfanyl)-1*H*-3-pyrrolyl]methanone **3b**


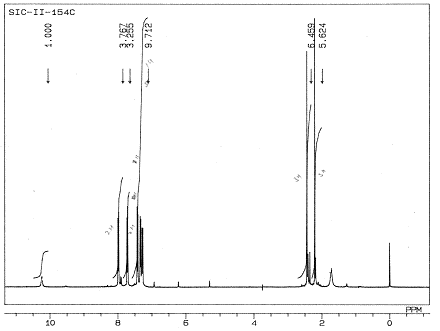


400 MHz (CDCl_3_) ^1^H NMR spectrum of (4-chlorophenyl)[5-[(4-methylphenyl)sulfonyl]-4-(methylsulfanyl)-1*H*-3-pyrrolyl]methanone **3c**


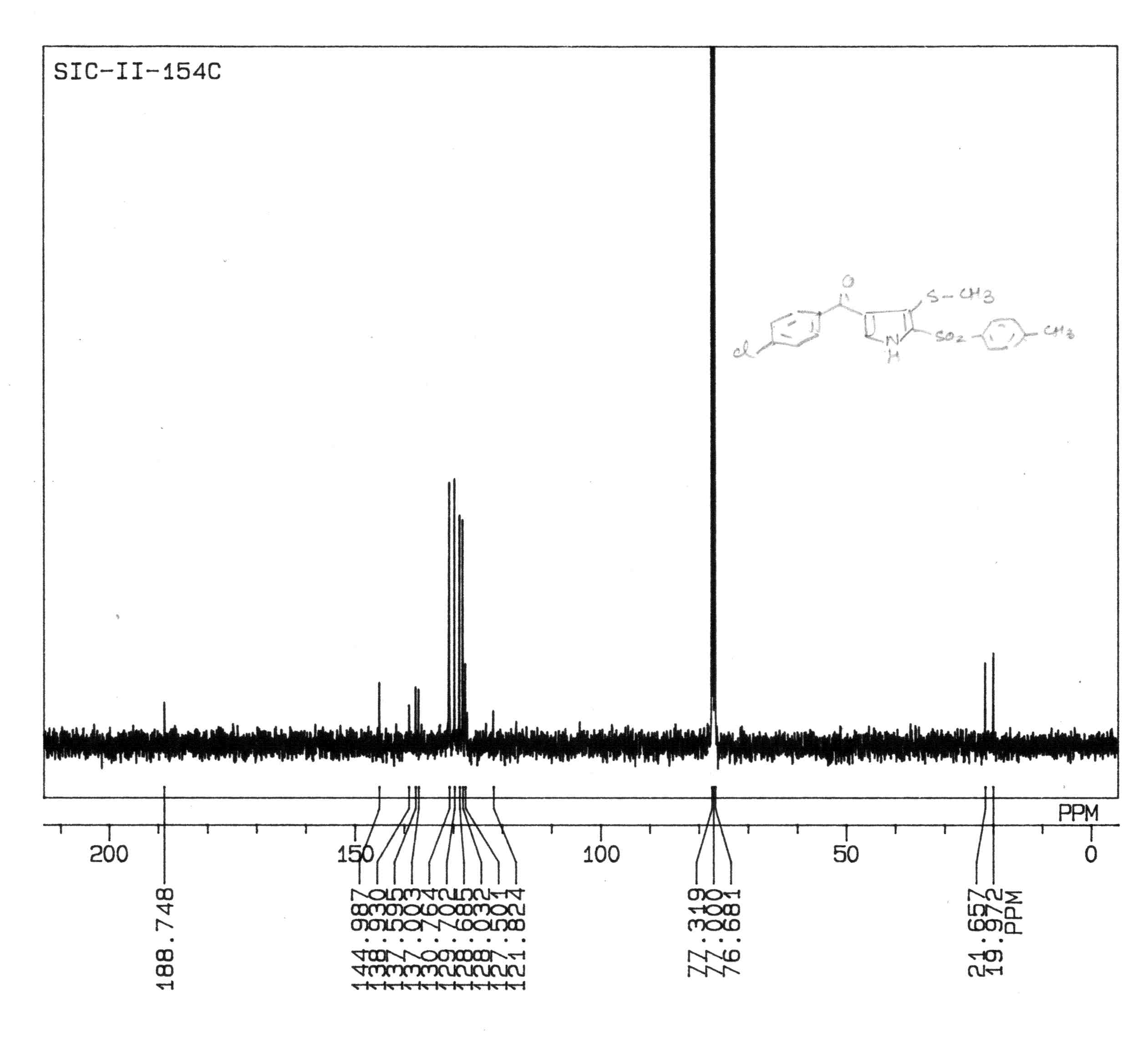


75 MHz (CDCl_3_) ^13^C NMR spectrum of (4-chlorophenyl)[5-[(4-methylphenyl)sulfonyl]-4-(methylsulfanyl)-1*H*-3-pyrrolyl]methanone **3c**


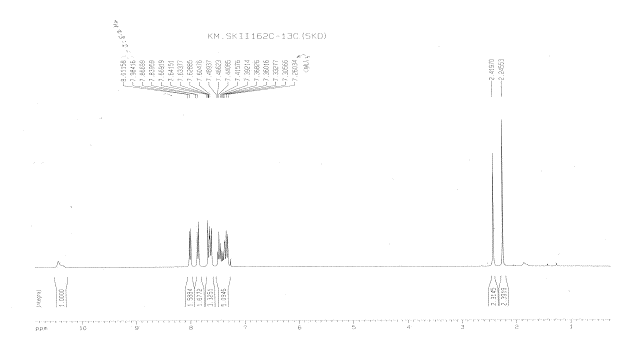


300 MHz (CDCl_3_) ^1^H NMR spectrum of (4-Biphenyl)[5-[(4-methylphenyl)sulfonyl]-4-(methylsulfanyl)-1*H*-3-pyrrolyl]methanone **3d**


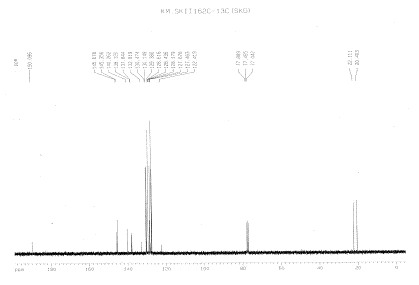


75 MHz (CDCl_3_) ^13^C NMR spectrum of (4-Biphenyl)[5-[(4-methylphenyl)sulfonyl]-4-(methylsulfanyl)-1*H*-3-pyrrolyl]methanone **3d**


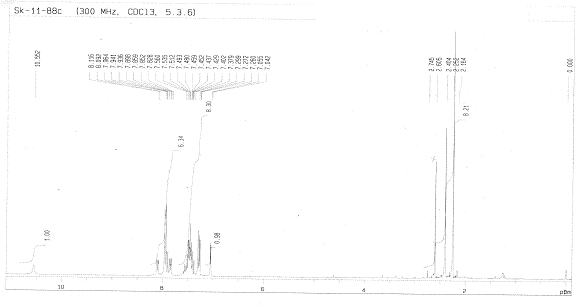


300 MHz (CDCl_3_) ^1^H NMR spectrum of [5-[(4-methylphenyl)sulfonyl]-4-(methylsulfanyl)-1*H*-3-pyrrolyl](1-naphthyl)methanone **3e**


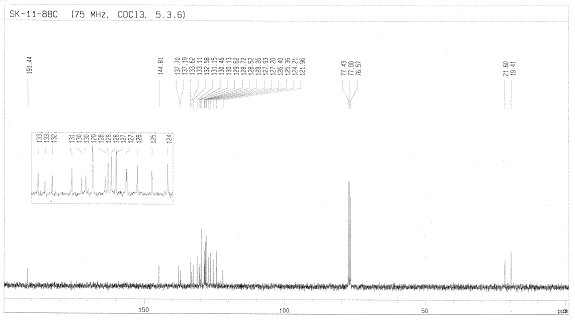


75 MHz (CDCl_3_) ^13^C NMR spectrum of [5-[(4-methylphenyl)sulfonyl]-4-(methylsulfanyl)-1*H*-3-pyrrolyl](1-naphthyl)methanone **3e**


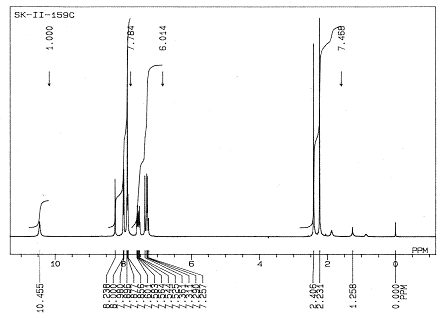


400 MHz (CDCl_3_) ^1^H NMR spectrum of [5-[(4-methylphenyl)sulfonyl]-4-(methylsulfanyl)-1*H*-3-pyrrolyl](2-naphthyl)methanone **3f**


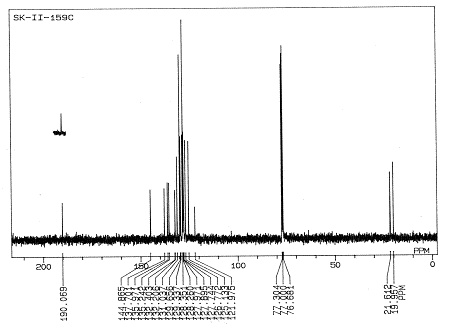


100 MHz (CDCl_3_) ^13^C NMR spectrum of [5-[(4-Methylphenyl)sulfonyl]-4-(methylsulfanyl)-1*H*-3-pyrrolyl](2-naphthyl)methanone **3f**

100 MHz (CDCl_3_) ^13^C NMR spectrum of [5-[(4-methylphenyl)sulfonyl]-4-(methylsulfanyl)-1*H*-3-pyrrolyl](2-naphthyl)methanone **3f**


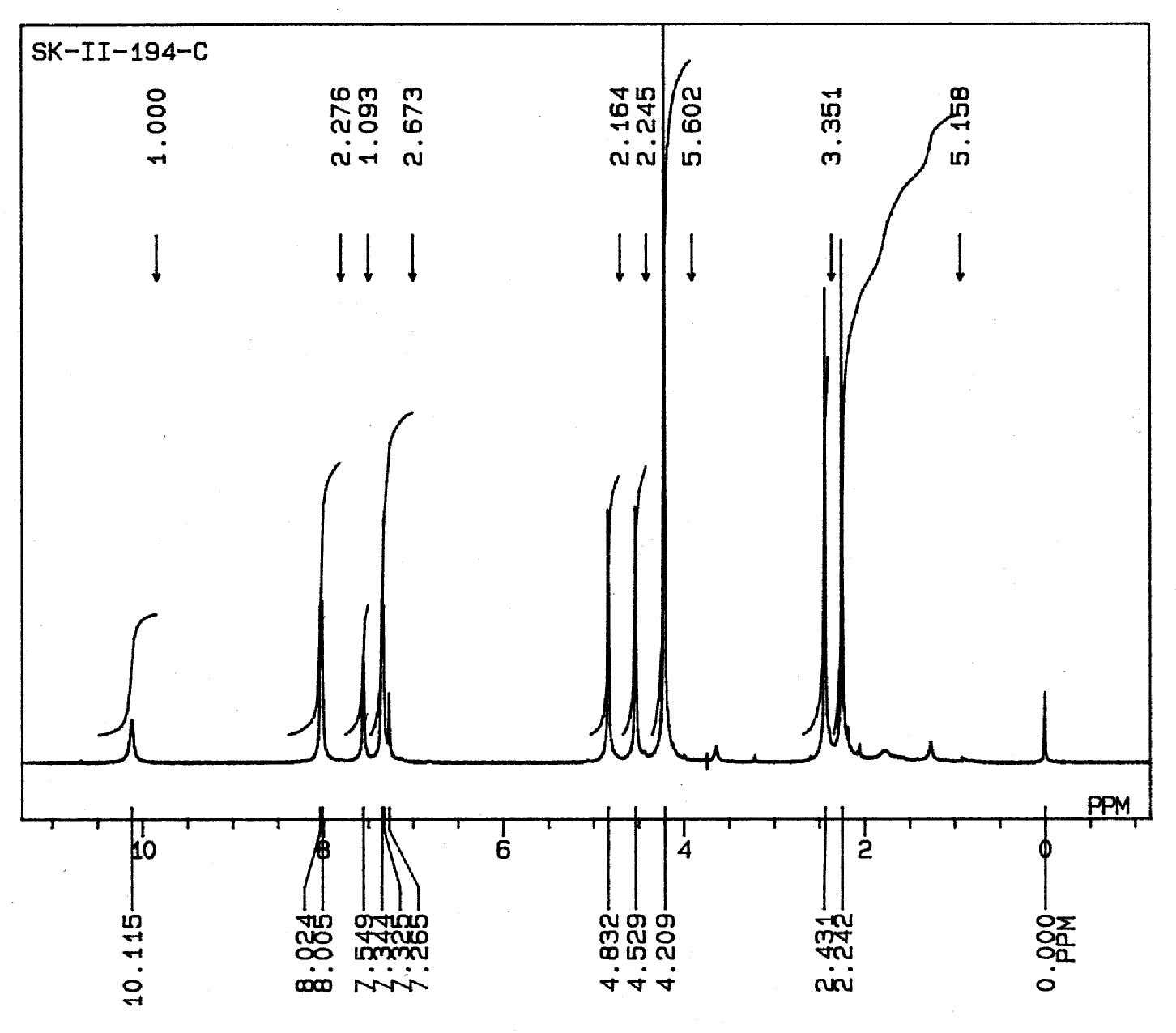


400 MHz (CDCl_3_) ^1^H NMR spectrum of [5-[(4-methylphenyl)sulfonyl]-4-(methylsulfanyl)-1*H*-3-pyrrolyl](3-η^5^-ferrocenoyl) methanone **3g**


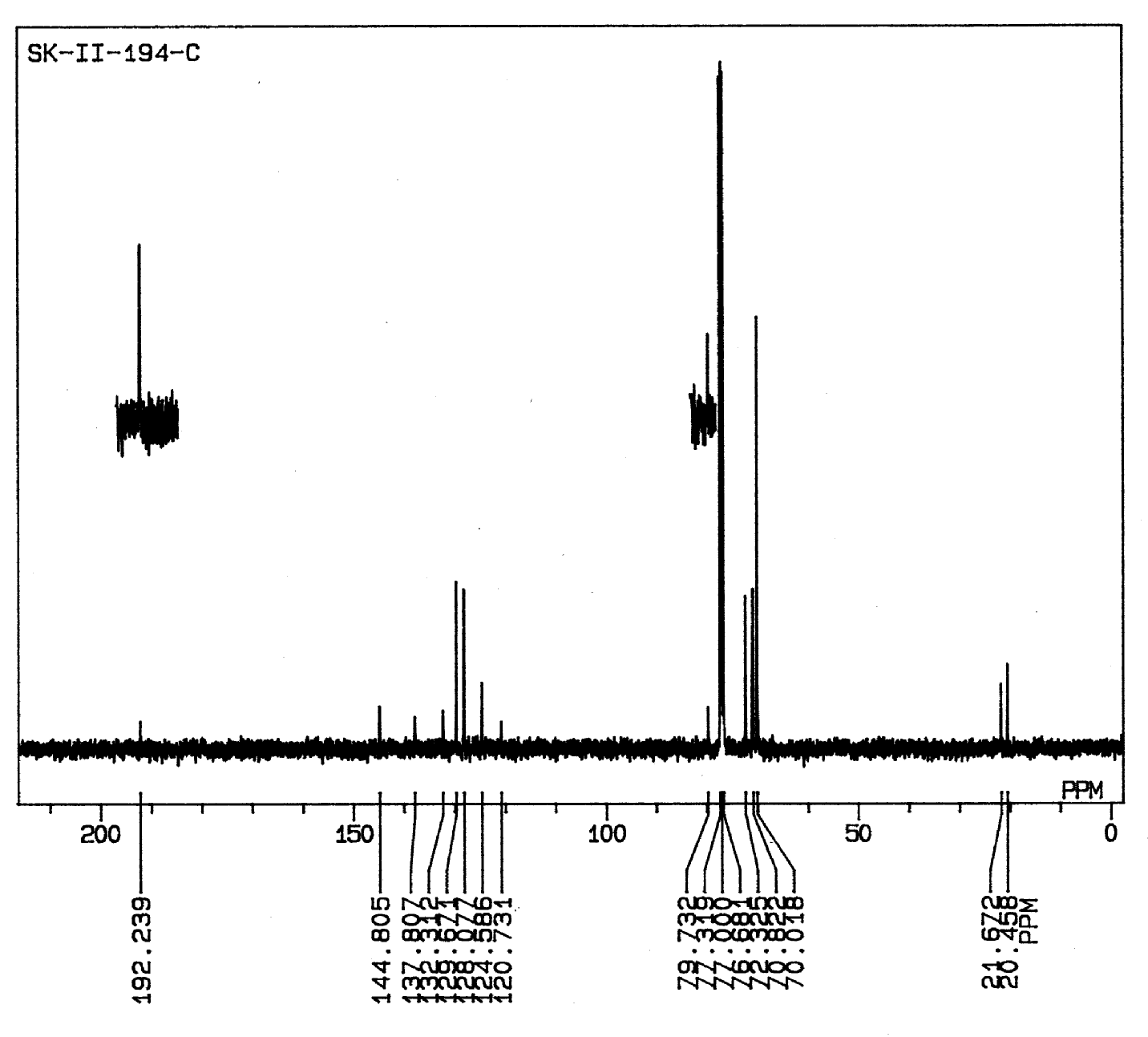


100 MHz (CDCl_3_) ^13^C NMR spectrum of [5-[(4-methylphenyl)sulfonyl]-4-(methylsulfanyl)-1*H*-3-pyrrolyl](3-η^5^-ferrocenoyl) methanone **3g**


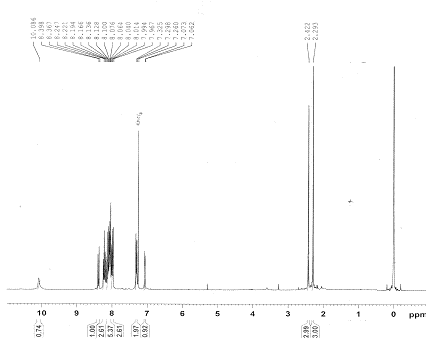


300 MHz (CDCl_3_) ^1^H NMR spectrum of [5-[(4-methylphenyl)sulfonyl]-4-(methylsulfanyl)-1*H*-3-pyrrolyl](1-pyrenyl)methanone **7**


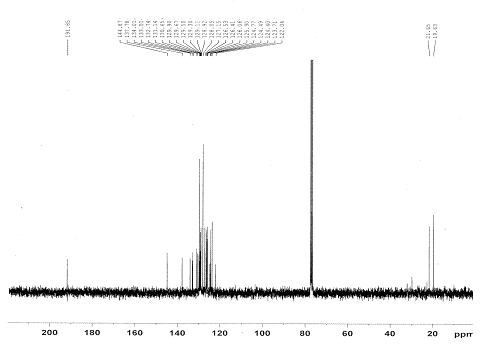


75 MHz (CDCl_3_) ^13^C NMR spectrum of [5-[(4-methylphenyl)sulfonyl]-4-(methylsulfanyl)-1*H*-3-pyrrolyl](1-pyrenyl)methanone **7**


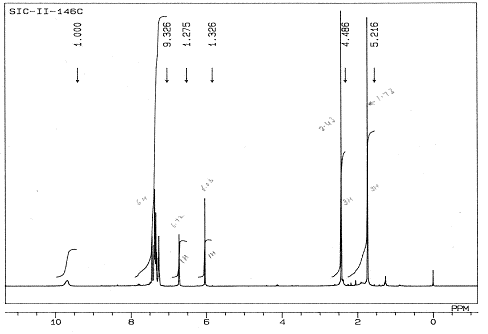


400 MHz (CDCl_3_) ^1^H NMR spectrum of 3,3-di(methylsulfanyl)-1-(4-phenyl-1*H*-3-pyrrolyl)-2-propen-1-one **9**


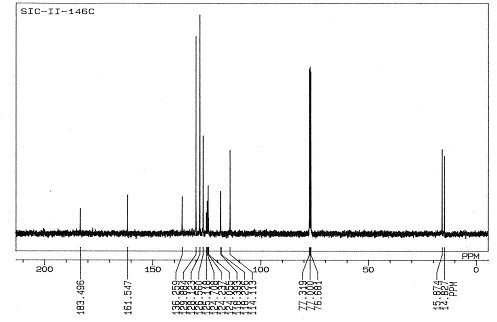


100 MHz (CDCl_3_) ^13^C NMR spectrum of 3,3-di(methylsulfanyl)-1-(4-phenyl-1*H*-3-pyrrolyl)-2-propen-1-one **9**
